# Comparison of methods for estimating the nucleotide substitution matrix

**DOI:** 10.1186/1471-2105-9-511

**Published:** 2008-12-01

**Authors:** Maribeth Oscamou, Daniel McDonald, Von Bing Yap, Gavin A Huttley, Manuel E Lladser, Rob Knight

**Affiliations:** 1 Department of Applied Mathematics, University of Colorado, Boulder, CO, USA; 2 Department of Computer Science, University of Colorado, Boulder, CO, USA; 3 Department of Statistics and Applied Probability, National University of Singapore, 21 Lower Kent Ridge Road 119077, Singapore; 4 John Curtin School of Medical Research, Australian National University, Canberra, Australia; 5 Department of Chemistry & Biochemistry, University of Colorado, Boulder, CO, USA

## Abstract

**Background:**

The nucleotide substitution rate matrix is a key parameter of molecular evolution. Several methods for inferring this parameter have been proposed, with different mathematical bases. These methods include counting sequence differences and taking the log of the resulting probability matrices, methods based on Markov triples, and maximum likelihood methods that infer the substitution probabilities that lead to the most likely model of evolution. However, the speed and accuracy of these methods has not been compared.

**Results:**

Different methods differ in performance by orders of magnitude (ranging from 1 ms to 10 s per matrix), but differences in accuracy of rate matrix reconstruction appear to be relatively small. Encouragingly, relatively simple and fast methods can provide results at least as accurate as far more complex and computationally intensive methods, especially when the sequences to be compared are relatively short.

**Conclusion:**

Based on the conditions tested, we recommend the use of method of Gojobori *et al*. (1982) for long sequences (> 600 nucleotides), and the method of Goldman *et al*. (1996) for shorter sequences (< 600 nucleotides). The method of Barry and Hartigan (1987) can provide somewhat more accuracy, measured as the Euclidean distance between the true and inferred matrices, on long sequences (> 2000 nucleotides) at the expense of substantially longer computation time. The availability of methods that are both fast and accurate will allow us to gain a global picture of change in the nucleotide substitution rate matrix on a genomewide scale across the tree of life.

## Background

The nucleotide substitution rate matrix, conventionally denoted *Q*, holds the rate of change from each type of nucleotide to each other nucleotide in the Markov model of molecular evolution [1] (see [2] for review). *Q *is often treated as a nuisance parameter in phylogenetic reconstruction, and is often assumed to be constant over a phylogenetic tree. However, if *Q *were really constant, all genomes would have identical composition, assuming that sufficient time had passed for the process to reach equilibrium. Neutrally-evolving sites reach equilibrium rapidly (e.g. within individual genera of nematodes [3]). However, genomes range in GC content (the fraction of nucleotides that are G or C, as opposed to A or T) from about 25% to 75% [4,5], and members of all three domains of life have a wide range of GC content [6]. Consequently, discovering how *Q *varies is key to building better phylogenies and to understanding fundamental processes of molecular evolution. However, there are many methods for providing an estimate of *Q*, i.e. $\hat{Q}$, that may differ substantially in accuracy and performance, and lack of information about these performance characteristics has hindered discovery of patterns of *Q *on a large scale within and between genomes. Several factors are known to influence the proportions of the four nucleotides in the genome (hereafter, genome composition), and hence presumably *Q*, under specific conditions. For example, in the spirochete *Borrelia burgdorferi*, the strand-specific compositional biases induced by deamination are so great that the strand on which each gene is coded can be determined simply by examining its composition [7,8]. Other examples include the influence of CpG doublets on mammalian gene composition [9], deamination biases in mitochondria [10], and the origin of mammalian isochores through variation in mutation patterns [11]. Within vertebrate genomes, the balance between oxidation and deamination mutations can also differ radically [12]. However, despite the potential for relating changes in *Q *to specific mutational processes, little attention has been paid to obtaining empirical $\hat{Q}$ in specific lineages [13,14].

That *Q *does vary, and that variations in *Q *can tell us something interesting about the underlying processes in molecular evolution, has thus drawn substantial attention only in the last 10–15 years. Most interest in obtaining $\hat{Q}$ has come from studies of phylogenetic reconstruction: for example, likelihood methods for tree-building typically involve optimizing a single $\hat{Q}$ matrix for a phylogenetic tree [15], and testing for the symmetries in $\hat{Q}$ that best explain the sequence data [16] is a standard part of modern phylogenetic inference workflows [17]. Although some work has been done on non-stationary models [18-23], and recent evidence suggests that non-stationarity may have a large influence on the accuracy of phylogenetic reconstruction [24,25], no comparison of the different methods for obtaining $\hat{Q}$ has yet been performed. Available methods for obtaining $\hat{Q}$ are based on different underlying assumptions, and are hence expected to differ both in accuracy and in speed of calculation. Most available methods obtain $\hat{P}$, where the entries of $\hat{P}$ are the estimated probabilities of changing from each base to each other base after a defined time interval of duration *t*. $\hat{Q}$ is related to $\hat{P}$ by the equation $\hat{P}=e^{\hat{Q}t}$. Therefore, $\hat{Q}$*t *can be obtained from $\hat{P}$ in two ways: either by simply taking the matrix logarithm of $\hat{P}$, or by using constrained optimization to find a valid $\hat{Q}$*t *without negative off-diagonal elements that, when exponentiated, produces $\hat{P}$ with minimal error. The time factor can be removed by scaling $\hat{Q}$*t *so that the trace is (-1), leaving $\hat{Q}$ on a standard scale (but with arbitrary units). For equilibrium processes, the weighted trace can be scaled such that $-\sum_{i}p_{ii}{\hat{Q}}_{ii}=1$, taking into account the equilibrium base frequencies [26] to standardize to a fixed number of substitutions per site, but for nonstationary processes this procedure is not justified. The simplest method for obtaining $\hat{P}$ is to count the frequencies that a base *i *∈ {*A*, *C*, *G*, *T*} in one sequence is aligned with base *j *∈ {*A*, *C*, *G*, *T*} in the other sequence, and denote this by *n*_*ij*_. To isolate the effects of mutation, regions of the sequence that are thought to be under no selection or very weak selection, e.g. introns or 4-fold degenerate synonymous sites, are used for this purpose. If the substitution process is time-reversible, *n*_*ij *_= *n*_*ji *_in expectation. It suffices to consider the special case where the observed counts *n *is symmetric. If *n *is not symmetric, we replace *n *by the average of *n *and the transpose *n'*[26]. Then the entries of $\hat{P}$ are given by ${\hat{P}}_{ij}=\frac{n_{ij}}{\sum_{k}n_{ik}}$, which can then be used to obtain $\hat{Q}$ as described above [2,26,27]. Alternatively, since this method of obtaining $\hat{P}$ assumes the divergence between sequences is small, $\hat{P}\sim I+\hat{Q}t$. Then an estimate for the rate of change of *i *to *j *is given by ${\left(\hat{Q}t\right)}_{ij}=\frac{n_{ij}}{\sum_{k}n_{ik}}$ for *i *≠ *j*, and the diagonal elements are set such that each row of $\hat{Q}$*t *sums to 0 [28,29]. We refer to this method as the 'Goldman' method [29]. Again the time factor can be removed by scaling $\hat{Q}$*t*. However, both of these methods are based on the assumption that the evolutionary process is time-reversible. A method that can better recapture non-time-reversible substitution processes uses rooted triples of sequences where X and Y are the sister taxa, and Z is the outgroup [30]. If X has the same base at that position as Z, the assumption is that the change most likely occurred between Y and its common ancestor with X. This method thus leads to a directional and not necessarily time-reversible $\hat{P}$, which can then be used to obtain $\hat{Q}$ (see [31] for additional details, and for a comparison of directional and undirectional estimates of *Q*). We refer to this three-sequence method as the 'Gojobori' method of otaining $\hat{P}$[30], and use the implementation in the PyCogent toolkit [32].

More elaborate methods include maximum likelihood methods and Markov triple analysis [13,33,34]. Each of these methods assume nucleotide sites are independent and identically distributed, and do not require the evolutionary process to be stationary or reversible (recall that the sequence composition produced by evolution according to a Markov process over time need not match the composition of the starting sequence, which might be modeled differently). To explain the methods, we introduce the following notation. Consider a rooted triple of sequences from taxa *X*, *Y*, *Z*. Define *R *to be the root and let ${\hat{V}}_{R}$ denote the estimated vector of nucleotide probabilities for the sequence at *R*. Let ${\hat{P}}_{U}$ denote the estimated nucleotide probability transition matrix from *R *to taxa *U*, for *U *∈ {*X*, *Y*, *Z*}, i.e.,

$$\begin{array}{ccc} {\hat{P}}_{U}(i,j)=Pr(U=j|R=i), & \text{for} & \begin{array}{cc} i,j\in \{A,C,G,T\}, & U\in \{X,Y,Z\}. \end{array} \end{array}\;$$

The transition probabilities in each row of ${\hat{P}}_{U}$ sum to 1, therefore each matrix ${\hat{P}}_{U}$ consists of 12 unknown parameters and similarly the vector ${\hat{V}}_{R}$ consists of 3 unknown parameters. To determine the unknown parameters, the methods each make use of the observed joint probability distribution between the three taxa. Let ${\hat{J}}_{X,Y,Z}$ denote this distribution. That is,

$$\begin{array}{ccc} {\hat{J}}_{X,Y,Z}(i,j,k)=Pr(X=i,Y=j,Z=k), & \text{for} & i,j,k\in \{A,C,G,T\}. \end{array}$$

Each state of ${\hat{J}}_{X,Y,Z}$ can be written in terms of ${\hat{P}}_{X}$, ${\hat{P}}_{Y}$, ${\hat{P}}_{Z}$ and ${\hat{V}}_{R}$. For example,

$$\begin{array}{ccc} {\hat{J}}_{X,Y,Z}(A,C,T) & = & \sum_{i\in \{A,C,G,T\}}Pr(R=i)Pr(X=A|R=i)Pr(Y=C|R=i)Pr(Z=T|R=i)\\ & = & \sum_{i\in \{A,C,G,T\}}{\hat{V}}_{R}(i){\hat{P}}_{X}(i,A){\hat{P}}_{Y}(i,C){\hat{P}}_{Z}(i,T). \end{array}$$

Maximum likelihood methods obtain ${\hat{P}}_{X}$, ${\hat{P}}_{Y}$ and ${\hat{P}}_{Z}$ by finding the values for the parameters that maximize the likelihood of the observed joint frequencies [33,34]. That is, align the *X*, *Y*, and *Z *sequences and let *n*_*XYZ*_(*i*, *j*, *k*) denote the number of times *X *= *i*, *Y *= *j*, *Z *= *k *occurs in the alignment. The likelihood function is proportional to the conditional probability, given by

(1) $$Pr(n_{XYZ}|P_{X},P_{Y,}P_{Z},V_{R})=\prod_{i,j,k\in \{A,C,G,T\}}{\hat{J}}_{X,Y,Z}{\left(i,j,k\right)}^{n_{\left(i,j,k\right)}}.$$

One method for finding the 39 unknown parameters (12 unknowns in each of three ${\hat{P}}_{i}$ and three unknowns in ${\hat{V}}_{R}$) is to use a global optimization algorithm to search parameter space and find ${\hat{P}}_{X}$, ${\hat{P}}_{Y}$, ${\hat{P}}_{Z}$ and ${\hat{V}}_{R}$ that maximize the likelihood. We refer to this method as the 'MW' method below. Since the chosen ordering of the nucleotides is arbitrary, there are 4! permutations of the elements of ${\hat{V}}_{R}$ (while keeping the nucleotide ordering fixed), and 4! corresponding permutations of the rows of the $\hat{P}$ matrices, that will result in the same ${\hat{J}}_{X,Y,Z}$ and therefore the same likelihood. Thus, once the global optimization algorithm converges to one of the maximum likelihood solutions, a unique solution can be determined by choosing the permutation that maximizes the sum of the traces of the ${\hat{P}}_{X}$, ${\hat{P}}_{Y}$ and ${\hat{P}}_{Z}$ matrices [34], which provides the solution that implies least change in the overall sequence (the diagonal elements represent the probability that a nucleotide remains unchanged after *t *time units). If the underlying process is not one that maximizes the diagonal entries, the process is unidentifiable using this method (i.e. cannot be recovered even with infinite sequence data). One solution might be to restrict the class of processes to those with diagonal-dominant *P *[35], although we did not perform such restrictions in this study.

An alternate approach for finding the maximum likelihood parameters that does not involve a global optimization routine was introduced by Barry and Hartigan and is referred to as the 'BH' method [33,34] below (we ran this method using default parameters as supplied). BH rewrites the likelihood function in terms of joint probability matrices along each edge of the tree. Solving for the maximum value of the likelihood leads to a system of iterative equations for the joint probability matrices along each edge. Thus, given initial starting guesses for the joint distributions along each edge, the system can be iterated until convergence is reached [33,34]. The joint probability matrices along each edge can then be used to obtain their corresponding $\hat{P}$ matrices.

BH is valid on unrooted trees of any size, but for simplicity we briefly outline it here using the unrooted version of the rooted triple defined above. Let *u*_*i *_denote the *i*th element in the sequence from taxon *U*, with *U *∈ *X*, *Y*, *Z*. Similarly, let *r*_*i *_denote the *i*th element in the sequence at node *R*. Then

$${\hat{J}}_{X,Y,Z}(x_{1},y_{1},z_{1})=\sum_{r_{1}\in \{A,C,G,T\}}{\hat{J}}_{X,R}(x_{1},r_{1}){\hat{P}}_{Y}(r_{1},y_{1}){\hat{P}}_{Z}(r_{1},z_{1}).$$

Let *N *denote the length of the sequences. Then the conditional probability can be written as

(2) $$Pr(x,y,z|{\hat{J}}_{X,R},P_{Y},P_{Z})=\prod_{i=1}^{N}\sum_{r\in \{A,C,G,T\}}{\hat{J}}_{X,R}(x_{i},r){\hat{P}}_{Y}(r,y_{i}){\hat{P}}_{Z}(r,z_{i}).$$

The maximum likelihood solutions are therefore the values of ${\hat{J}}_{X,R}$, ${\hat{P}}_{Y}$ and ${\hat{P}}_{Z}$ that maximize the likelihood subject to the constraint that the entries in ${\hat{J}}_{X,R}$ sum to 1, as it is a joint probability matrix. In other words, BH maximizes

$$\log\left(Pr(x,y,z|{\hat{J}}_{X,R},{\hat{P}}_{Y},{\hat{P}}_{Z})\right)+\lambda \left(1-\sum_{x,r\in \{A,C,G,T\}}{\hat{J}}_{X,R}(x,r)\right).$$

Differentiating with respect to ${\hat{J}}_{X,R}$ and *λ *and setting equal to zero leads to the following iterative equation for ${\hat{J}}_{X,R}$,

$${\hat{J}}_{X,R}(x,r)=\frac{1}{N}\sum_{i=1}^{N}\frac{I(x\in \{x_{i}\}){\hat{J}}_{X,R}(x,r){\hat{P}}_{Y}(r,y_{i}){\hat{P}}_{Z}(r,z_{i})}{\sum_{r\in \{A,C,G,T\}}{\hat{J}}_{X,R}(x,r){\hat{P}}_{Y}(r,y_{i}){\hat{P}}_{Z}(r,z_{i})},$$

where *I*(*x *∈ {*x*_*i*_}) is an indicator function that takes the value 1 if *x *= *x*_*i *_and 0 otherwise [33,34]. This iterative equation can be written entirely in terms of joint distributions along each edge by using the relation

(3) $$\begin{array}{ccc} {\hat{P}}_{U}(r,u_{i})=\frac{{\hat{J}}_{U,R}(u_{i},r)}{\sum_{u_{i}\in \{A,C,G,T\}}{\hat{J}}_{U,R}(u_{i},r)}, & \text{for} & U\in \{X,Y,Z\}. \end{array}$$

These steps are repeated to derive iterative equations for ${\hat{J}}_{Y,R}$ and ${\hat{J}}_{Z,R}$, leading to an iterative system for ${\hat{J}}_{X,R}$, ${\hat{J}}_{Y,R}$ and ${\hat{J}}_{Z,R}$. Suitable initial values are chosen and the system is iterated until it converges [33].

The $\hat{J}$ matrices can then be converted to $\hat{P}$ matrices along each edge using the relationship in (3). Finally, the $\hat{P}$ matrices can then be used to obtain $\hat{Q}$ matrices using the relationship $\hat{P}=e^{\hat{Q}t}$.

Both the global optimization and BH methods maximize the same likelihood function, but they differ in the computational methods used to find the maximum and in whether they write the unknown parameters in terms of joint probability matrices or probability transition matrices. Maximizing the likelihood does not result in a unique solution without adding further restrictions. MW chooses the $\hat{P}$ matrices with the largest sum of the traces, while in the BH method, the initial starting values determine which maximum is chosen. The BH method's default initial $\hat{J}$ matrices, are diagonally dominant. Thus we expect the iteration to converge to a diagonally dominant solution (if one exists). Thus, in theory the BH and MW methods will result in the same $\hat{Q}$ matrices. In practice, their answers can differ if either converge instead to a local maximum, or if the initial starting points used in either algorithm lead to too slow of a rate of convergence, or if the original *P *matrices used to generate the sequences are not diagonally dominant.

Another method, which we refer to as the 'Lake' method, uses Markov triple analysis (MTA) as a different approach to finding the ${\hat{P}}_{U}$ matrices for rooted triples of sequences [13]. This method uses the fact that the conditional joint probabilities ${\hat{J}}_{X,Y|Z}$ can be written in terms of ${\hat{P}}_{X}$, ${\hat{P}}_{Y}$, and ${\hat{P}}_{Z}^{*}$, where ${\hat{P}}_{Z}^{*}$ is the probability transition matrix from taxon *Z *to *R*. (Note that because the evolutionary process is not required to be reversible, ${\hat{P}}_{Z}^{*}$ differs from ${\hat{P}}_{Z}^{*}$.) For example,

$${\hat{J}}_{X,Y,|Z}(A,C|T)=\sum_{r\in \{A,C,G,T\}}{\hat{P}}_{Z}^{*}(T,r){\hat{P}}_{X}(r,A),{\hat{P}}_{Y}(r,C).$$

MTA uses the observed conditional joint probabilities as estimates of the true conditional joint probabilities. This results in a system of 48 nonlinear equations in terms of the 36 unknowns in ${\hat{P}}_{X}$, ${\hat{P}}_{Y}$ and ${\hat{P}}_{Z}^{*}$. MTA solves this system by first using the properties of determinants to set up a simpler system in terms of ${\hat{P}}_{Z}^{*}$. Specifically, determinants of the conditional joint probabilities are used to derive a system of 6 quartic polynomials. ${\hat{P}}_{Z}^{*}$ is determined by first finding the roots of the polynomials and then searching through 24^5 ^possible orderings of the roots to find the ordering that results in a consistent system. In the case that the original system of 48 equations is inconsistent, the Lake method chooses the ordering that minimizes an inconsistency function defined by the author [13]. These ordered roots are coefficients in a system of equations that is linear in the unknown parameters of ${\hat{P}}_{Z}^{*}$. Thus once the ordered roots are found, ${\hat{P}}_{Z}^{*}$ can be determined by solving the linear system. Again, any of the 4! permutations of the columns of ${\hat{P}}_{Z}^{*}$ will also be a valid solution. Thus the Lake method chooses the ordering with a positive determinant that maximizes the trace. Once a unique ${\hat{P}}_{Z}^{*}$ has been determined, 3 systems of equations that are linear in the parameters of ${\hat{P}}_{X}$, ${\hat{P}}_{Y}$, ${\hat{P}}_{Z}$ can be solved to determine these matrices. Again, the $\hat{P}$ matrices estimated using the Lake method can then be used to obtain $\hat{Q}$ matrices.

If the observed conditional frequencies do in fact accurately estimate the true conditional frequencies and result in a consistent system of 48 equations, the Lake method provides a computationally feasible way to find the solutions of the system. However, since this method estimates the joint probability distribution from observed frequencies, the accuracy of this estimation will be dependent on sequence length and sequence alignment. Therefore, the system of 48 equations can often be inconsistent (i.e. values for the 36 parameters that satisfy all of the equations and that are between 0 and 1 do not exist). In this case, it is possible that the Lake method will be unable to find valid estimations of the probability transition matrices, as the linear systems in the method may also be inconsistent or may result in ${\hat{P}}_{X}$, ${\hat{P}}_{Y}$ or ${\hat{P}}_{Z}$ having negative elements.

In this paper, we present a comparison of the speed and accuracy of the different methods described above, using sequences of different lengths and divergences. We also compare two methods for obtaining $\hat{Q}$ matrices from the resulting $\hat{P}$ matrices: using the matrix log [36] and using constrained optimization.

## Results and discussion

In principle, each of the methods described has advantages and disadvantages. The Goldman method uses the observed number of changes between two sequences to obtain $\hat{Q}$. It is thus very simple and fast computationally. However it assumes that the evolutionary process is time-reversible and that the divergence between sequences is small, which may not model real evolutionary processes well. The Gojobori method obtains $\hat{P}$ from groups of three taxa in which the outgroup is known, by counting the directed changes in two sister taxa relative to the outgroup, then normalizing the rows of this count matrix. It does not require the evolutionary process to be time-reversible and thus is more general than the Goldman method. Since both the Goldman and Gojobori methods rely on the count matrix, we would expect them both to do better on longer sequences, which provide more observed data for more accurately sampling $\hat{P}$ and $\hat{Q}$. The maximum likelihood methods (MW and BH) do not require the evolutionary process to be stationary or reversible, and in principle should be more robust to sampling error in the observed count matrix. However, maximizing the likelihood alone does not lead to a unique solution for the $\hat{P}$ matrices without imposing further restrictions. By default, these methods resolve this issue by searching for the diagonally dominant solution. Thus, we expect these methods to do the best when the original *Q *matrices are such that their corresponding *P *matrices are diagonally dominant for the level of sequence divergence under consideration.

Finally, the Lake method uses observed conditional joint probabilities as estimates for the true conditional probabilities. If the observed probabilities accurately estimate the true probabilities and result in a consistent system of equations, the Lake method should provide an accurate estimate of *Q*. However, because this method depends on solving several linear systems that may be inconsistent on the given data, it is the only method of those we consider that, in certain cases, fails to provide any valid output for $\hat{P}$. We would expect it to do worse on shorter sequences, when the observed conditional joint probabilities estimate of the true joint probabilities poorly due to sampling error. Overall, although some of the methods are restricted in their solution space, our primary interest is in determining empirically how accurately each method obtains $\hat{Q}$ from a randomly generated *Q*.

### Different techniques differ by orders of magnitude in performance, and the fastest techniques are among the most accurate

The different techniques for obtaining $\hat{P}$, and hence $\hat{Q}$, vary widely in both accuracy and performance. We measured accuracy by testing for the ability to recover *Q *from three-taxon trees with specified topology and branch length using the standard Markov model of evolution and arbitrary, randomly generated substitution matrices with no constraints on symmetry or the relative sizes of the elements. Error was measured using the Euclidean distance between the parametric *Q *that the sequences were evolved under and the inferred $\hat{P}$ for the same sequences.

**Figure 1:**
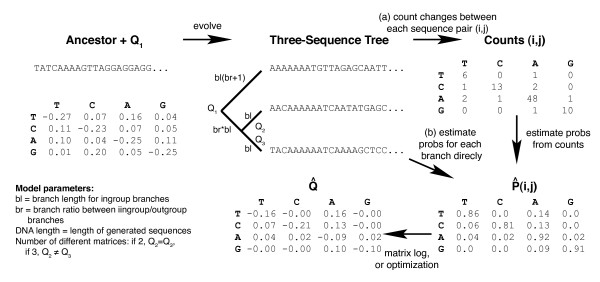
**Overview of the workflow**. The overall workflow is as follows. First, one or more random rate matrices (depending on parameter settings) is generated, and sequences evolved according to a three-taxon tree of known topology. These sequences are generated as an ungapped alignment, so alignment issues do not affect the result (i.e. we assume that the alignment process is perfect). Depending on the algorithm, we then either (a) infer counts that differentiate specific pairs of sequences, then infer the probability matrices for different branches by normalizing the count matrices, or (b) infer the probability matrices for different branches directly from the alignment. Finally, the rate matrices are inferred from the probability matrices (either by the matrix log method or the constrained optimization method), normalized to eliminate the time component, and compared against the original rate matrix or matrices used to generate the sequences. Parameters that we varied during the analysis are marked on the tree and shown in the bottom-left of this figure.

The speed and accuracy of each method were tested under the following conditions. The length of the sequence ranged from 100 to 1000 in steps of 100, then 1000 to 5000 in steps of 1000 (inclusive). The length of the inner branch ranged from 1 to 10% divergence in steps of 1%, and from 10 to 50% divergence in steps of 5%. The branch ratio, i.e. the ratio of the inner branch to the outer branch, was 0.1 to 1 in steps of 0.1, and 1 to 10 in steps of 1. The substitution matrix was either kept constant for the two inner branches, or varied between these two branches. We sampled 100 random matrices under every possible combination of these conditions. The overall workflow, including a description of these parameters, is shown in Figure 1 (adapted from [31]). Because we are studying nonstationary processes, we scaled the trace to (-1) (i.e. we did not weight by the equilibrium base frequencies), but this procedure is valid for the purposes of comparing the methods and was used consistently for all methods.

**Figure 2:**
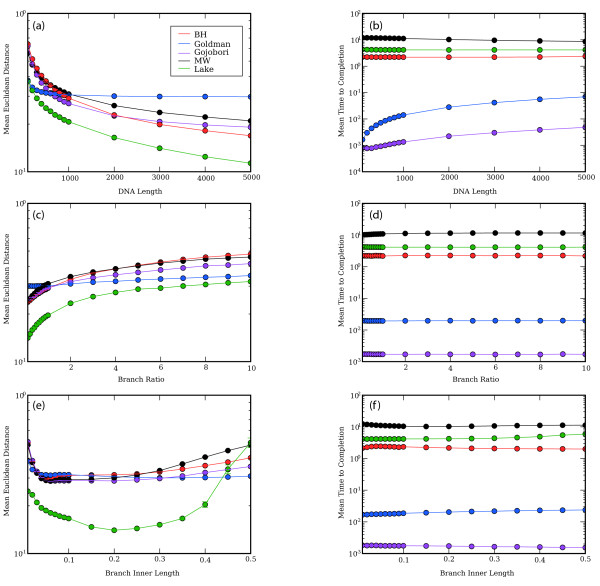
**Results considering all matrices**. Results for accuracy (left column) and speed (right column) for the different methods (legend in panel a) under different simulation conditions. Error is measured as the mean Euclidean distance between the matrix used to generate the sequences and the inferred matrix (arbitrary units); time is measured in seconds. Figures show effects of DNA length (a, b), branch ratio (c, d) and branch inner length (e, f) on accuracy and speed. The effect of whether the inner branches evolved according to the same or different matrices was negligible, and is not shown.

Figure 2 summarizes the speed and accuracy of the different methods. Each point shows the average for all points where the condition holds, e.g. the point for sequence length 100 averages over all the trials in which the sequence length was 100 nucleotides. Whether the two inner matrices were the same or different had negligible effect on either the speed or accuracy of the methods (data not shown, results shown are averages over both conditions). The Lake method appears to be most accurate under a wide range of conditions, although this is an artifact of its inability to operate on data generated using matrices that produce inaccurate results using all methods (see the next section below). The Gojobori and Goldman methods ran up to 3 orders of magnitude faster than the other methods, and had comparable or better accuracy (specifically, the Goldman method outperformed the others at short sequence lengths, less than 600 nucleotides, and the Gojobori method performed well at longer sequence lengths). In general, there was no clear association between speed and accuracy: the MW method is among the slowest methods but not generally among the more accurate methods under the conditions tested, for example, whereas the Gojobori method is generally the fastest and has accuracy very similar to methods that it outperforms by orders of magnitude. However, the BH method is most accurate on very long sequences (over 2000), although it is much slower than either the Gojobori or Goldman method in this range. One cautionary note about the MW method is that we reduced the maximum number of iterations from 1000 to 100, and increased the tolerance from 10^-10 ^to 10^-5^, to improve run-time. Using the default parameters might increase the accuracy at an additional substantial runtime penalty, although even under the conditions tested this method is too slow for large-scale applications.

### The apparent benefits of the Lake method are due in large part to its failure to run on difficult datasets

**Figure 3:**
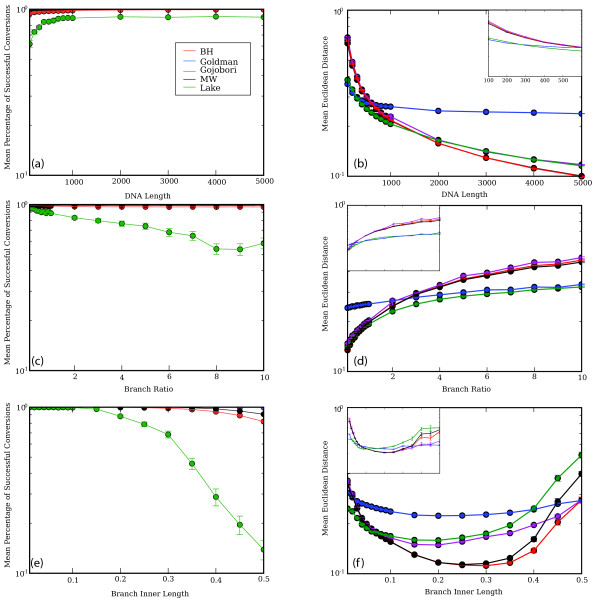
**Results showing the subset of alignments on which the Lake method completed successfully**. Results for probability of completion (left column) considering all matrices, and accuracy (right column) considering only the subset of matrices for which the Lake method completed, for the different methods (legend in panel a) under different simulation conditions. Completion is measured as the fraction of trials in which a given method completed; error is measured as the mean Euclidean distance between the matrix used to generate the sequences and the inferred matrix (arbitrary units). Figures show effects of DNA length (a, b), branch ratio (c, d) and branch inner length (e, f) on ability to complete successfully and accuracy respectively. Insets in panels b, d, and f show the subset of the data where the branch lengths were below 600 nucleotides, where the Goldman method performed relatively well: scale is the same as the main panel in each case, except for b (where the length ranges from 100 to 600 nucleotides, as shown). The effect of whether the inner branches evolved according to the same or different matrices was negligible, and is not shown.

From the data in Fig. 2, it appears that the Lake method is more accurate than most other methods under most conditions. Because the Lake method is one of the slower methods, and also fails to produce results on a substantial fraction of matrices, we decided to test whether this apparent increase in accuracy was due to the method rejecting datasets for which the other methods produced especially inaccurate predictions. Figure 3 demonstrates that this is the case: when we consider only the subset of datasets on which the Lake method was able to complete successfully, we find that the results of the Lake method are worse than the results of other methods under many conditions, and are only better in a minority of cases where the branch ratio (ratio between inner and outer branches) is very long, the sequence is short, or the inner branch length is short. For these reasons, and because the method fails to complete on as many as 80% of the datasets under some conditions, we believe that the apparent advantages of this method do not outweigh its disadvantages relative to other methods.

### The two methods for converting probability matrices to rate matrices performed indistinguishably

We compared two different methods for converting the probability matrices to rate matrices: simply taking the matrix logarithm, and constrained optimization of the rate matrix to ensure that the resulting rate matrices contained only non-negative off-diagonal elements (a common numerical issue with rate matrix inference: negative off-diagonal elements have no meaning). The accuracy results (i.e. the Euclidean distance between parametric and inferred matrices collected under each condition) were visually and statistically indistinguishable, and the values from 265 randomly sampled points did not differ significantly in mean error (two-sample *t *test, *p *=0.53). We performed an additional internal control by testing whether the estimates of the two inner branches' rate matrices were equivalent when the two inner branches were simulated under the same *Q *with similar results (*p *= 0.49, *n *= 265). Matrices with negative off-diagonal elements were not corrected in any way: however, the distances between these matrices with negative off-diagonal elements and the corresponding matrices corrected by constrained optimization were apparently equivalent. This result is not necessarily unexpected because most negative off-diagonal elements produced by taking the logarithm of the *P *matrix are small compared to the negative elements on the diagonal.

## Conclusion

In general, fast methods provided little or no degradation of accuracy relative to slower methods under the conditions tested: either the Gojobori method or the Goldman method, which were orders of magnitude faster than the other methods, gave equivalent or improved accuracy under most conditions. Overall, we recommend the Gojobori method when the sequences are long (at least 600 nucleotides), the Goldman method for shorter sequences, and the BH method for very long sequences (over 2000 nucleotides) if computation time is not an issue. We did not observe improved accuracy (as measured by Euclidean distance between the matrices) using constrained optimization rather than the matrix logarithm for obtaining $\hat{P}$ matrices from $\hat{Q}$ matrices, but constrained optimization does have the desirable property that the resulting matrices have only non-negative off-diagonal elements (the results shown are averaged over all matrices, whether or not they have negative off-diagonals). One cautionary note about the work presented here is that we examined random rate matrices: as data are collected about which kinds of rate matrices are biologically reasonable, it may be useful to revisit this study using biologically-inspired rate matrices, which may reveal larger differences among methods.

The findings presented here open the door to very large-scale studies of the variation in nucleotide substitution matrices in different genes and taxa, which would be completely impractical with the slower methods. We can thus begin to explore the evolution of this fundamental parameter in all models of molecular evolution, and perhaps ultimately discover the molecular basis for differences in nucleotide substitution patterns in different organisms.

## Methods

The implementations of the methods we used for the comparison were as follows: the MTA algorithm was implemented in C by Michael Newton; the BH algorithm was implemented in Java, written by V. Jayaswal, L. Jermiin, J. Robinson as provided at the following website: [33]; the MW method was implemented in C by Michael Woodhams using simulated annealing as the optimization routine; and the Gojobori and Goldman methods were implemented in Python and contributed to PyCogent [32]. Simulations were run on a 200-core Opteron cluster using PBS-Torque and custom scripts. Inferred rate matrices with negative off-diagonal elements were used directly for error calculations. All programs were run with default parameters unless otherwise described: for the BH method, we set the number of iterations to 100. Random rate matrices were generated using PyCogent's random rate matrix generation capabilities: specifically, a vector of uniformly distributed random numbers between 0 and 1 is generated for the off-diagonal elements of each row, the diagonal element is set such that the row sums to 1, and the entire matrix is normalized so that the trace is -1.

## Authors' contributions

MO wrote the code to run the simulations, implemented the Goldman method and performed the bulk of the research. DM wrote code to run the simulations, and performed some of the data analysis and assisted with technical issues. GAH and VBY implemented the constrained optimization method. GAH, MEL, and RK directed the research. MO and RK wrote the manuscript.
